# Mid-Infrared Electrochromics Enabled by Intraband Modulation in Carbon Nanotube Networks

**DOI:** 10.1021/acsami.2c19758

**Published:** 2023-02-17

**Authors:** Peter J. Lynch, Manoj Tripathi, Aline Amorim Graf, Sean P. Ogilvie, Matthew J. Large, Jonathan Salvage, Alan B. Dalton

**Affiliations:** † Department of Physics and Astronomy, University of Sussex, Brighton BN1 9RH, U.K.; ‡ School of Pharmacy and Biomolecular Science, University of Brighton, Brighton BN2 4GJ, U.K.

**Keywords:** carbon nanotubes, electrochromics, emissivity modulation, optoelectronics, liquid processing

## Abstract

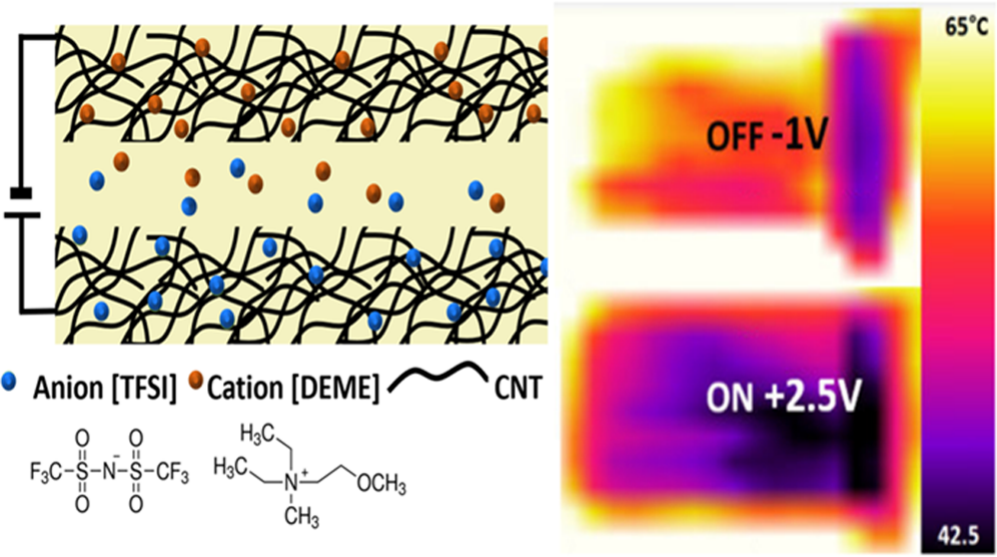

Tuneable infrared properties, such as transparency and emissivity, are highly desirable for a range of applications, including thermal windows and emissive cooling. Here, we demonstrate the use of carbon nanotube networks spray-deposited onto an ionic liquid-infused membrane to fabricate devices with electrochromic modulation in the mid-infrared spectrum, facilitating control of emissivity and apparent temperature. Such modulation is enabled by intraband transitions in unsorted single-walled carbon nanotube networks, allowing the use of scalable nanotube inks for printed devices. These devices are optimized by varying film thickness and sheet resistance, demonstrating the emissivity modulation (from ∼0.5 to ∼0.2). These devices and the understanding thereof open the door to selection criteria for infrared electrochromic materials based on the relationship between band structure, electrochemistry, and optothermal properties to enable the development of solution-processable large-area coatings for widespread thermal management applications.

## Introduction

Electrochromism, the phenomenon by which biasing a material changes an optical characteristic, opens up a variety of applications from the visible to the infrared (IR). Visible electrochromics can be used for narrow band coloration or as broadband “curtains”.^1,2^ IR electrochromics, in particular, could enable smart windows to modulate solar heat capture or release as a significant proportion of solar radiation is in the near-infrared (NIR, 0.8–2.5 μm) region. Additionally, mid-infrared (MIR, 2.5–25 μm) coincides with the black body spectrum of objects between 30 and 100 °C, this amount a hot body radiates at these temperatures in this frequency window is related to the emissivity. Electrochromism in the MIR window would allow for tuning of emissivity of objects to enable thermal control in environments, where convective cooling is more challenging such as in outer space applications.

Battery-type electrochromic devices allow ions under bias to move through an electrolyte and charge the respective electrodes to induce the reversible optical modulation. Due to the requirement for two electrodes, both electrode materials would ideally be electrochromic for a device of modulated transmission although this is not a requirement for nontransparent devices of modulated reflection. The most studied cathodic and anodic materials for electrochromics are WO_3_^3−6^ and NiO,^7−11^ respectively. These materials have broadband changes in optical properties from the visible to the IR due to the intercalation of ions, which produces an intercalated compound with modified band structure.^12^ With such comprehensive work on established materials, the current research is mostly focused on the stability and switching time. Alternative materials include V_2_O_5_,^13^ Nb_2_O_5_^14,14^ and conjugated polymers.^15^ These materials generally exhibit broadband visible-to-NIR response with particular attention paid to the visible.

Recently, nanocarbon-based materials, especially graphitic structures, have been utilized as electrochromic materials for emissivity modulation. The Dirac cone band structure of graphene allows shifting of the Fermi level by an external bias and results in Pauli blocking of optical transitions.^16^ These devices can be considered supercapacitor-type analogues to the battery-type electrochromics. Graphene prepared by chemical vapor deposition (CVD) has been shown to enable electrochromic modulation from the terahertz (THz) range to the visible.^16−18^ The MIR effect has also been demonstrated using filtered reduced graphene oxide (rGO) films^19^ and in multiwalled carbon nanotube (MWCNT) sheets.^20^ This shows that this modulation is robust to modification of the band structure of graphene due to defects or structure. Previous works have used either CVD materials (multilayer graphene or matted MWCNT sheets) or require hazardous chemical treatments (e.g., hydrazine treatment for GO to rGO^21^). In addition, CVD graphene requires a transfer process to an appropriate substrate, which adds another process along with polymer and metallic residues.^22^

Liquid-phase exfoliation (LPE) is a top-down approach for processing powders of bulk graphite or bundled carbon nanotubes (CNTs) into dispersions of few-layer nanosheets or individualized tubes. This is achieved by either selecting appropriate stabilizers in water or a solvent with suitable surface energy. Complementarily, centrifugation strategies can be used for controlled selection of the dispersion properties.^23,24^

By producing a liquid dispersion, one can then tailor the properties for a wide range of printing processes. LPE has been used to produce nanomaterial inks for inkjet, spraying, and screen-printing methods. In particular, chirality-selected nanotubes have been used to produce all-printed NIR notch filters.^25,26^ These notch filters focus on the semiconducting van Hove singularity transition in nanotubes.^27^ This follows research into band-filling in carbon nanotubes to elicit visible,^28,29^ NIR,^29,30^ and MIR optical responses. Vacuum-filtered films of double-walled CNTs have been used to confer electrochromic capabilities in the MIR and THz regime.^31^ The advantages of these printed films include the ability to conform to arbitrary substrates, including surfaces with compound curvature, and patterned devices to bestow additional functionality, such as grating or metasurface structures.

In this work, we report a symmetrical electrochromic device using single-walled carbon nanotubes (SWCNTs) spray-deposited onto an ionic liquid-infused membrane. We develop an understanding of the SWCNT band structure as well as film thickness properties to produce a device with electrochromic properties in the MIR, which also operates as a device with variable emissivity.

## Results and Discussion

**Figure 1:**
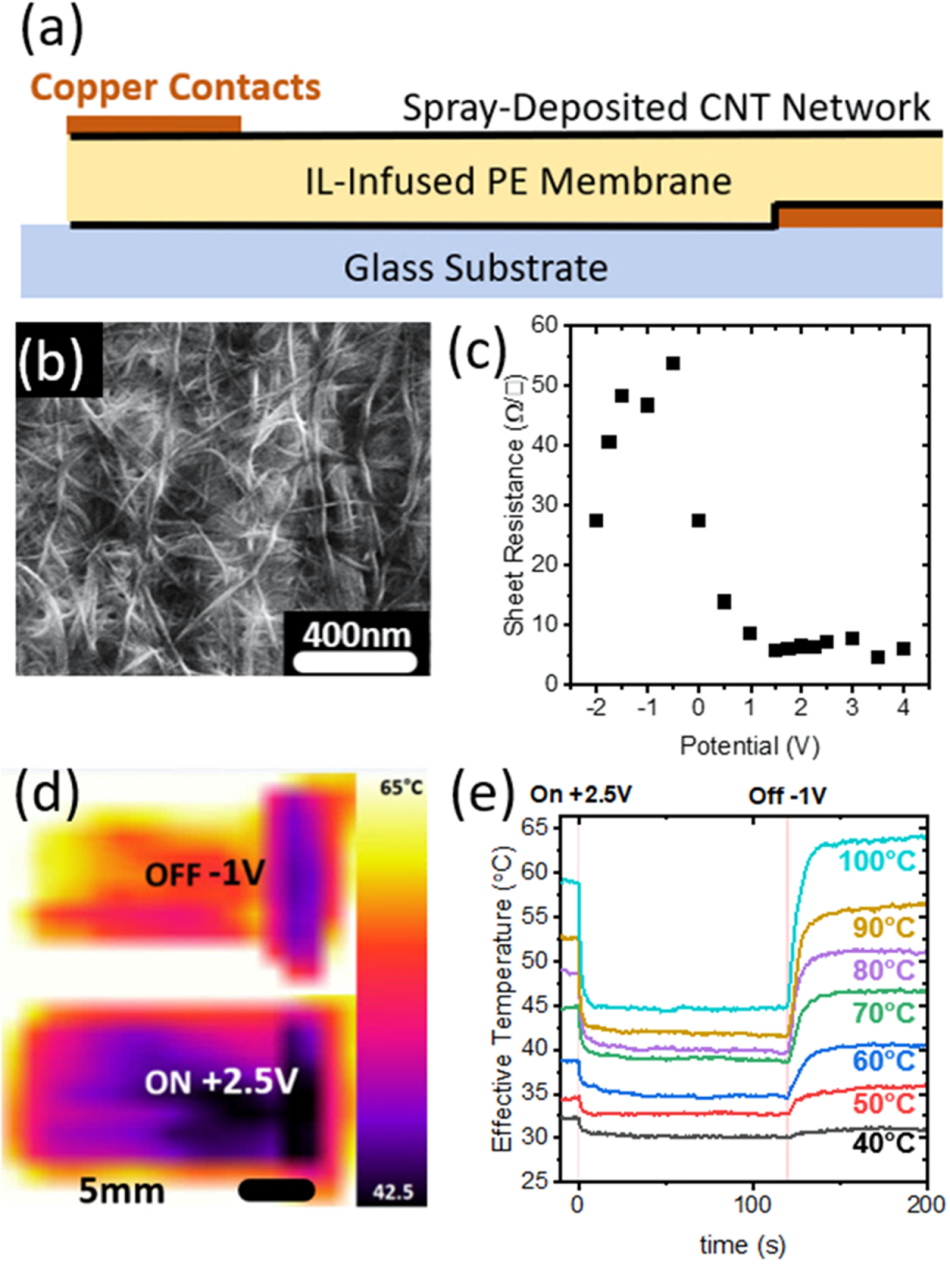
(a) Schematic of the device. Spray-deposited CNT network on either side of an ionic liquid-infused polyethylene membrane. The device is contacted on opposite sides and ends with copper tape before being attached to a glass substrate. (b) SEM on the spray-deposited CNT network on an uninfused PE membrane to demonstrate the morphology and porosity of the film. (c) Variation of sheet resistance of top electrode of the device measured by the four-point probe method under biasing from −2 to 3 V. (d) Images from a thermal camera of a device on a 100 °C hotplate in the off state (−1 V, appearing warmer) and the on state (2.5 V, appearing colder). (e) Time–temperature profiles of the same device showing a change in effective temperature as observed by a thermal camera at varying hotplate temperatures from 40 to 100 °C, the device is at 0 V for *t* < 0 s and is switched to +2.5 V for 0 s < *t* < 120 s and down to −1 V from *t* > 120 s.

Devices were fabricated as supercapacitor-type electrochromic structures by spray deposition of carboxymethyl cellulose (CMC)-stabilized SWCNTs on an ionic liquid-infiltrated polyethylene (IL/PE) membrane (see Materials and Methods Section). The SWCNT networks were deposited uniformly on both sides to ensure impedance matching as close as possible to improve performance.^32^Figure 1a depicts the structure of the device. Under bias, anions and cations are preferentially displaced to the cathode and anode, respectively. A scanning electron micrograph of a sprayed film (Figure 1b) shows the morphology of these nanotubes on a polyethylene membrane in a practical realization. It is a laterally isotropic network of nanotubes showing connectivity well above the areal percolation threshold. There are large bundles visible on the surface with nanotubes conforming to the porous membrane beneath. The porous nature of these networks allows the unimpeded flow of ions necessary to prevent mass transport limitations in electrochemical devices. Analysis of the data shows single-walled tubes with diameters as low as 0.5 nm and bundles up to 20 nm in diameter (Figures S1 and S2).

The biasing of these devices causes the Fermi level to shift and has been shown to modulate electronic and optical properties in carbon nanotubes.^25^ The sheet resistance (Figure 1c), determined from four-point probe measurements on the top surface of the device, shows appreciable changes with applied bias, and this is analogous to an electrolyte-gated field-effect transistor (FET) device, where the back electrode acts as the gate.^33^ This suggests that there will be corresponding modulation of the MIR properties as the resistance change is indicative of Fermi level shifting, which blocks optical transitions in nanocarbons.^17,25^Figure 1d shows the device on a hotplate at 100 °C, initially biased at 0 V before 0 s, then switched to +2.5 V in the on state (near the minimum sheet resistance), and then biased at −1 V in the off state (near the maximum sheet resistance in Figure 1c) under a thermal infrared camera with observed temperatures going from over 60 °C to below 50 °C. The time–temperature profile of the device at a variety of hotplate temperatures is shown in Figure 1e. Approx. 90% of the temperature drop occurs over <10 s when slewing from 0 to +2.5 V and similarly going into the off state of −1 V. The temperature drop is more pronounced as the temperature increases due to the emissivity switching effect. Emitted energy is related to the emissivity and the difference in the fourth power of the temperatures. It is also interesting to note that there is an inversion in the performance of the device in the on and off states between 40 and 50 °C, possibly indicating temperature-dependent population of states.

**Figure 2:**
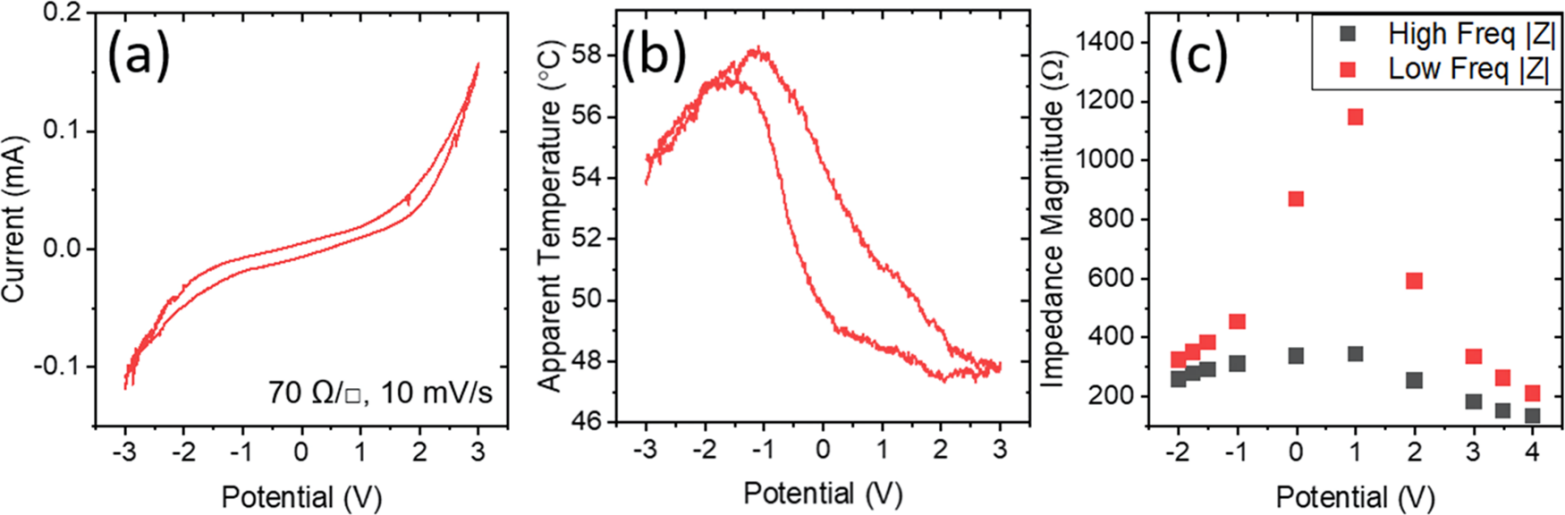
(a) Cyclic voltammetry of a 70 Ω/□ device at 100 °C swept between −3 and +3 V at 10 mV/s. (b) Temperature as observed by thermal camera of the previous device under cycling. (c) Impedance of the device at high (100 kHz) and low (0.2 Hz) frequency limits.

As the device is a supercapacitor-type structure, electrochemical characterization, via cyclic voltammetry at 10 mV/s, provides insights into the device operation. Figure 2a,b shows the current density and apparent device temperature, as observed by an IR camera while the device is on a 100 °C hotplate, respectively. The current density between −3 and +3 V is asymmetric, with larger currents achieved at the corresponding positive potential compared to the negative potential. Slight broadenings beyond the traditional non-Faradaic box-like shape of a supercapacitor indicate changes in the carrier density near the shifted Fermi level of the device.^34,35^ There are also characteristics of electrolysis (above 2 V and below −2 V) due to the device being run in an ambient environment. The electrolyzed species are most likely water, which narrows the potential window of ionic liquids and affects electrical properties,^36,37^ and oxygen dissolved, which is stable in ionic liquids albeit with low diffusivity.^38^ The effective temperature of a device at 100 °C shows a maximum of 57 °C at around −1 V and a minimum at +2 to 3 V. A hysteresis is noted, which could be due to degradation of the electrode under the higher potentials, and this is explored further in Figures S3 and S4.

Impedance spectroscopy was performed to determine the impedance of the device at high- and low-frequency limits. Figure 2c shows the impedance of the device at various bias potentials. The low-frequency limit is dominated by capacitive and/or faradaic processes that shows a dramatic decrease in resistance in either direction from a maximum at +1 V bias. This maximum appears at positive bias rather than at negative as the device resistance is a convolution of both electrodes and the bulk electrolyte resistance. The high-frequency limit is dominated by the resistive components contributed by the top and bottom electrodes, plus the electrolyte while the low frequency includes the impedance contribution from the capacitive reactance and charge transfer resistance. The high resistance in the low-frequency range indicates a minimum in faradaic reactions occurring in the device indicating an optimum potential for the electrolyte.

**Figure 3:**
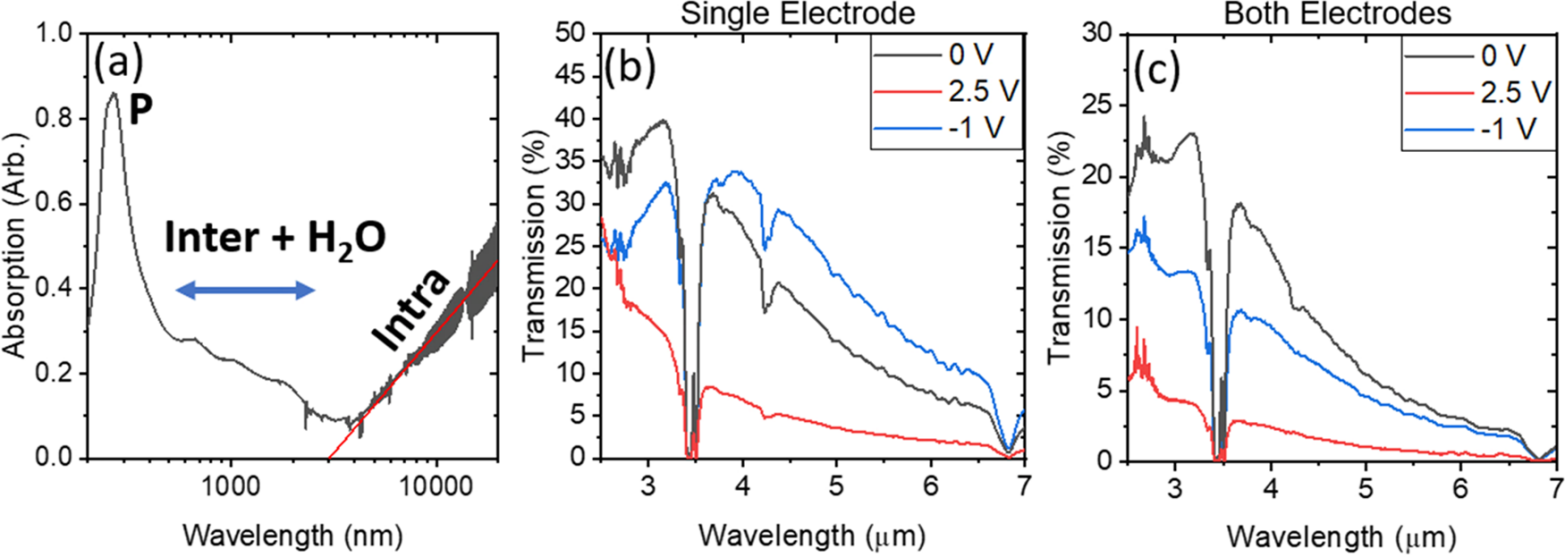
(a) Stitched UV to MIR spectrum of CNT film plasmon, interband and water absorptions and intraband absorptions annotated P, inter + H_2_O, and intra, respectively. (b) Transmission of the device where beam path passes through only one electrode (sheet resistance = 7 Ω/□) when the device is unbiased at +2.5 V and at −1 V. (c)Transmission of the device where beam path passes through both electrodes (sheet resistance = 13 Ω/□) when the device is unbiased at +2.5 and at −1 V.

The devices presented are modeled on electrochromic systems, and it is well understood that thermal emissivity and infrared optical properties are inextricably linked. For nontransparent materials, emissivity can be related to the absorption of the material by the equation ε = *A* = 1 – *R*, where ε, *A*, and *R* are emissivity, absorption, and reflection, respectively. However, the devices in this study are partially transparent, further complicating the analysis of emissivity. For these reasons, FTIR spectroscopy was used to analyze devices of varying SWCNT film thickness. This is due to the effects that band occupancy has on the dielectric constant and, thus, optical properties of graphitic materials.^16^Figure 3a shows the optical to infrared spectrum achieved through stitching of ultraviolet (UV)–visible data for liquid samples and NIR-MIR spectra of films on polyethylene. Interestingly, the absorbance of nanotubes increases on either side of a minimum at 3 μm wavelength. At longer wavelengths, intraband transitions are observed for semiconducting nanotubes, as a consequence of the Fermi level being within the valence band as reported by Wu et al.^27^ This has been attributed to oxygen doping from air^39^ and could also be due to the presence of a charged surfactant^40^ and could be responsible for the asymmetric electrochemical behavior of the device. Fitting through the scatter originated from interference due to film and substrate thickness (red line Figure 3a) shows a logarithmic relationship possibly due to an overlap of band structure behavior and the polydispersity of the tubes deposited. Below 3 μm wavelength, the excitonic transitions of van Hove singularities of the SWCNTs S_11_ (first semiconducting excitonic transition), S_22_ (second semiconducting excitonic transition), and M_11_ (first metallic excitonic transition) absorption peaks are observed in the order of decreasing wavelength and increasing energy^27^ though, due to the presence of hygroscopic CMC, the possible interference of water absorption can obscure these peaks. At higher energies, the plasmon absorption feature is observed.

Figure 3b shows the MIR transmission properties of a single film of SWCNT biased to potentials corresponding to the unbiased (0 V), on state (+2.5 V, blue-shifted) and off state (−1 V, red-shifted). The large, broad shifts suggest that this is dominated by electrode effects rather than chemical changes in the electrolyte. This device was constructed as the normal sandwich devices but with an aperture masked on one of the electrodes such that radiation only passed through one of the SWCNT layers. For the most part, this device has low to zero transparency in the MIR region, with the exception of a peak around 3 μm. The sharp feature around 3.3 μm is an absorption feature of the IL/PE substrate, namely, polyethylene C–H stretches (Figure S5). This is due to the Fermi level being below the S_11_ band gap.^27^ It is to be noted that with positive bias, transmission generally decreases and blueshifts occur, while at negative bias, a redshift is observed. These changes can be attributed to shifting the Fermi level in the region beyond the van Hove singularity, which has a *E*^–0.5^ dependence in the density of states.^27^Figure S6 shows the MIR properties of all measured single-film devices. Figure 3c shows the transmission spectrum of a standard device in the unbiased, on, and off states such that the radiation is passing through both SWCNT films on either side of the membrane. The transmission properties of each electrode in the unbiased state are effectively the same as the single-film device. However, due to each electrode having an opposite bias, the transmission becomes a combination of both electrodes, each oppositely shifted. The transmission decreases with bias and shifts in the position of the maxima are less pronounced due to a corresponding bias occurring on the counter electrode. FTIR spectra of all devices under bias are included in Figure S7.

The relative performance of films of varying sheet resistance can be analyzed by applying the understanding developed to investigate transparent electrodes.^41^ Transparent conductors can have the resistance transmission relationship defined by the following equation
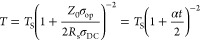
1where *T* is the device transmittance at a given wavelength, *T*_S_ is the substrate transmittance, *Z*_0_ is the impedance of free space ≈ 377 Ω, *R*_S_ is the sheet resistance of the conductive film, σ_op/DC_ are the optical and direct current conductivities, α is the material absorbance, and *t* is the thickness. Sheet resistance in this formulation can be thought of as a proxy measure for the film thickness since the two are linked by the direct current conductivity by *t* = 1/(*R*_s_σ_DC_). This proxy is used because the measurement of the thickness of a thin film on a porous surface is experimentally challenging. Though, on a flat substrate, well over the percolation threshold, the CNT film has a conductivity of 386,000 S/m (Figure S8). The dimensionless conductivity ratio σ_op_/σ_DC_ is conventionally treated as a figure of merit (FoM) for performance since increasing values indicate a film which has an increasing transmittance at a given sheet resistance (or conversely a lower sheet resistance at a given transmittance).

**Figure 4:**
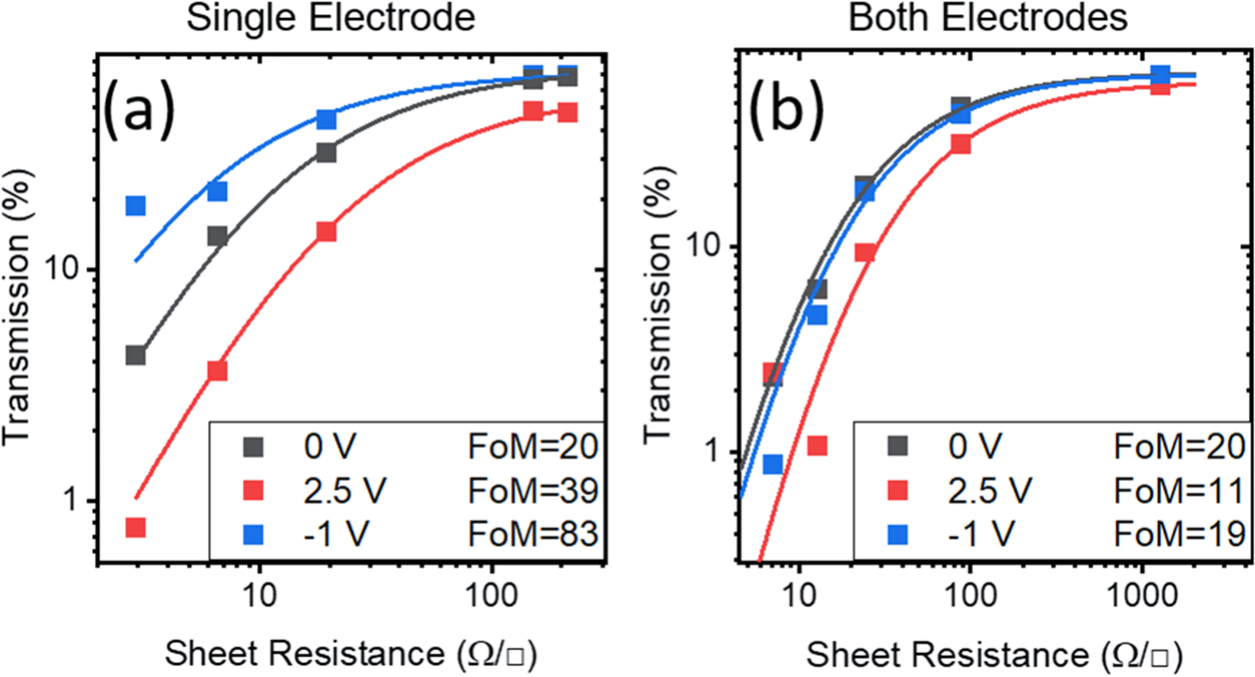
(a) Transmission at 5 μm of devices with beam path passing through only one electrode of varying sheet resistances from 1.3 to 220 Ω/□ at 0, 2.5, and −1 V with the corresponding fitting of TR curve. (b) Transmission at 5 μm of devices with beam path passing through both electrodes of varying sheet resistances from 7 to 1050 Ω/□ at 0, 2.5, and −1 V with the corresponding fitting of TR curve.

Figure 4a shows the transmittance of various films at 5 μm wavelength as a function of sheet resistance of the films at 0 V. It is noted that the sheet resistance of the films changes with bias and as such fitting the data with eq 1 yields the change in absorbance of the nanotube layers at that particular wavelength. In this case, the relative change in absorbance is from 83.3 in the off state to 19.6 in the on state corresponding to a fourfold increase in absorption of the nanotube layer.

The optoelectronic properties of the standard device can be modeled with a similar formulation to eq 1, except that the bracketed term has an exponent of −4 rather than −2 to represent the two sequential films in the device stack as represented in eq 2.
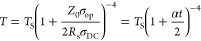
2Figure 4b shows the fitting of the transmittance and sheet resistance of two-layer devices, which result in a device absorbance change of up to a factor of 2. This is less than the single-layer device due to the opposite bias producing contending modifications to the optoelectronic performance on each electrode.

**Figure 5:**
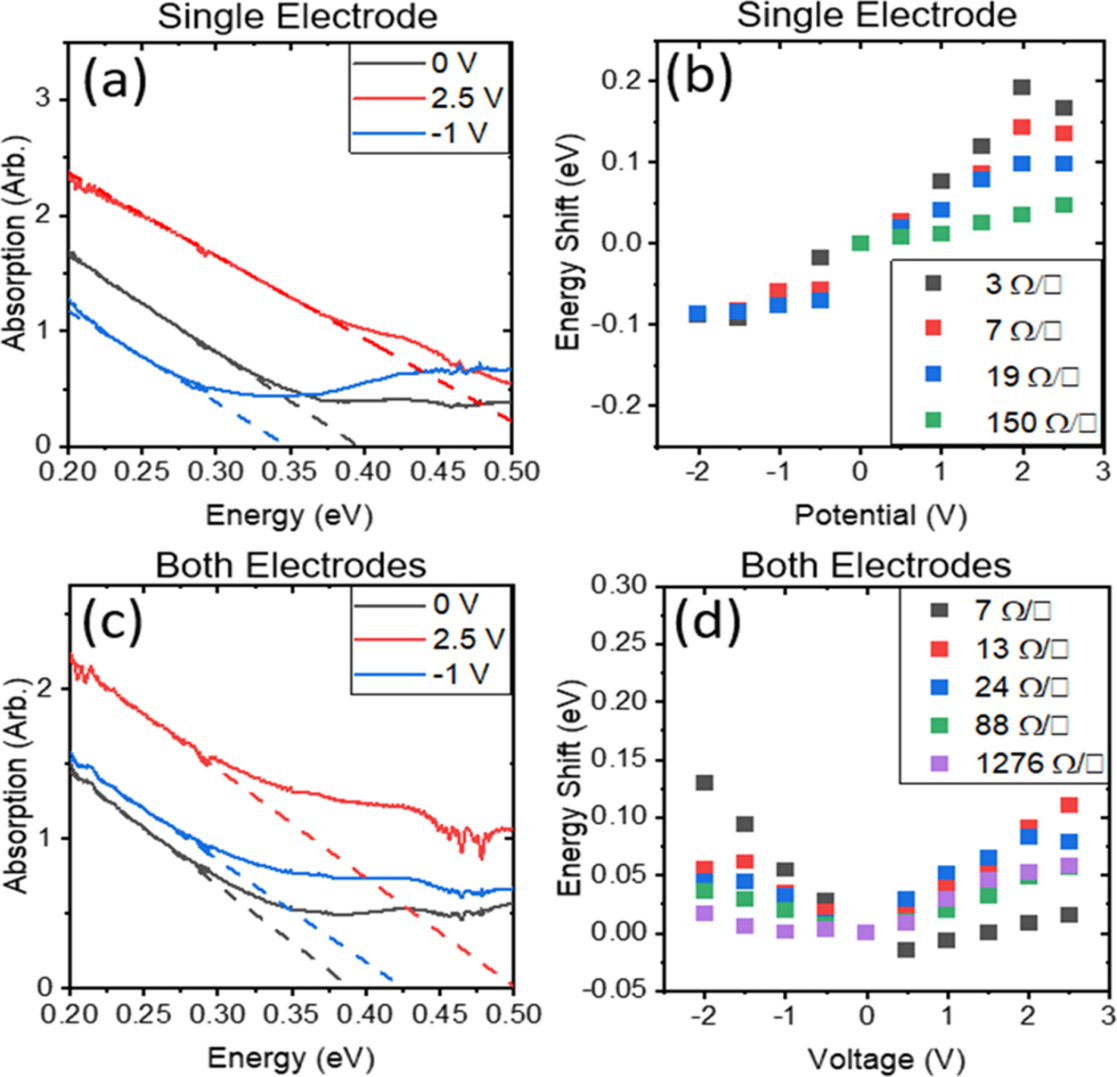
(a) Absorption spectrum plotted against photon energy to determine the intercept of the intraband absorption feature of the device of 7 Ω/□ with beam path going through only one electrode at 0, 2.5, and −1 V. (b) Observed energy shift from 0 V state of devices with beam path passing through only one electrode of varying sheet resistances from 1.3 to 19 Ω/□ at −2 to +2.5 V. (c) Absorption spectrum plotted against photon energy to determine the intercept of the intraband absorption feature of the device of 13 Ω/□ with beam path going through both at 0, 2.5, and −1 V. (d) Observed energy shift from 0 V state of devices with beam path passing through only one electrode of varying sheet resistances from 7 to 1276 Ω/□ at −2 to +2.5 V.

By subtracting the absorbance of the substrate, one is left with the absorbance of the carbon nanotubes, CMC, and residual electrolyte. The position of the band edge can be extracted through fitting the linear trend of the absorbance near the band edge, and this is illustrated for single-layer devices in the unbiased, on and off states in Figure 5a with all spectra shown in Figure S9. This shift in transmission change in the MIR has been studied previously using chemical doping of nanotubes,^42^ noting that doping that influences the excitonic transitions correspondingly alters Drude-type intraband transitions in the MIR. The band edge positions for unbiased films are in the range of 2.9 (0.43 eV) and 3.5 μm (0.35 eV). A redshift is observed on negative bias and a blueshift is observed under positive bias as seen in Figure S9. The films of different sheet resistances and the band positions under bias are shown in Figure 5b.

For standard devices (Figures 5c,d and S10), regardless of bias, a blueshift is observed, and this is due to the asymmetry of the band structure. The energy shifts observed tend to be in the range of thermal energy around 50 °C (approx. 0.025 eV), which may explain the asymmetry in the shifting behavior observed in Figure 1e.

Finally, the effect of bias on the apparent temperature and effective emissivity was investigated. Emissivity of a body is represented as a fraction of the radiation emitted by a black body, and this property for opaque bodies is effectively 1 – *R*, where *R* is the reflectivity in the MIR for bodies between room temperature and 100 °C. For transparent materials, it is closely related to the absorption of the body. Effective emissivity is a convolution of the radiation emitted from the SWCNT surface and the IR radiation that passes through it from the body underneath. The body underneath in this case is glass, which has an emissivity very close to 1. Figure S11 shows the switching of devices on to +2.5 V and off to −1 V at a range of temperatures similar to Figure 1e.

**Figure 6:**
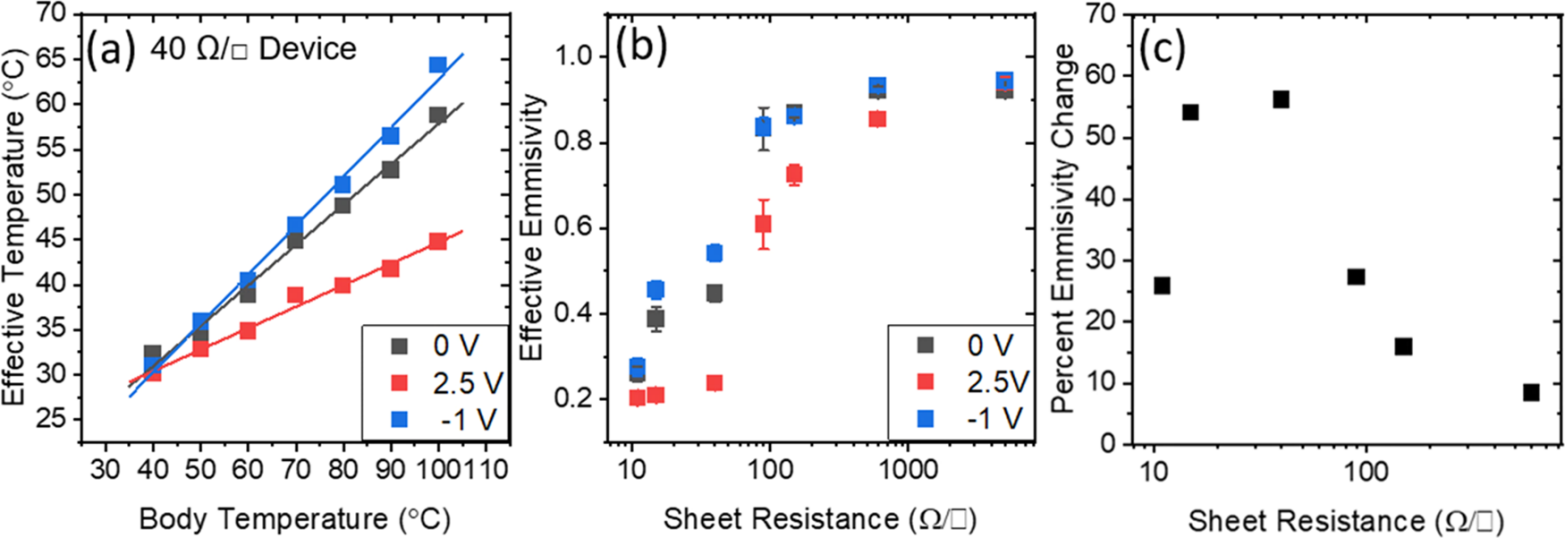
(a) Effective temperature for 40 Ω/□ device under biases of +2.5, 0, and −1 V at various body temperatures from 40 to 100 °C. (b) Effective emissivity vs sheet resistance as derived from linear fits of apparent temperature vs body temperature for devices of various sheet resistances. (c) Emissivity change expressed as a percentage of the difference between the maximum (at −1 V) and minimum (at +2.5 V) emissivities divided by the maximum emissivity.

The effective emissivity is estimated by fitting a line through the device temperatures at different body temperatures and biases. Figure 6a demonstrates this fitting for the 40 Ω/□ device. Further fittings for the other devices are shown in Figure S10. By plotting the emissivity at different bias potentials and against sheet resistance, one can metricize the effectiveness of these materials for emissivity modulation. Figure 6b shows the effective emissivities for various states of operation with Figure 6c, quantifying the percent emissivity change as a function of sheet resistance. This leads to the conclusion that devices of around 50 Ω/□ yield the biggest relative change in emissivity losing over 50% of the effective emissivity between the off and on states. As the sheet resistance decreases, the effective temperature of the device also decreases. The percent emissivity change (Δε = ε_off_ – ε_on_/ε_off_, where ε_on_ and ε_off_ are the emissivities in the on and off states, respectively) however, increases (Figure 6c) to a maximum and decreases below a certain sheet resistance. This is due to SWCNT films themselves having a very low emissivity, and a large part of the emissivity is coming from the substrate below and the ionic liquid.

To contextualize the results with respect to carbon-based materials in the literature, data for similar electrochromic devices is presented in Table 1. The devices are categorized in terms of the device structure, potential range, and emissivity characteristics. It is notable that the state-of-the-art in terms of performance is the MWCNT material drawn as a sheet from a CVD reactor, which shows one of the highest emissivities in the off state and the lowest emissivity in the on state. The work with our SWCNTs compares favorably by having similar performance to the CVD graphene materials in terms of relative performance but suffers from having a lower emissivity in the off state. However, our work outperforms the liquid-processed devices in the literature in both range of emissivity and general processability. The processability of these devices is significantly easier and cheaper than devices based on CVD techniques as spraying can be made compatible with roll-to-roll and other industrial processes. Furthermore, with advances in other printing techniques, resistance limitations in electrodes could be countered by patterning low-resistance current collector meshes of the same material.

**Table 1:** Properties of Carbon-Based IR Emissivity Modulating Devices in the Literature

front electrode	*R*_s_ (Ω/□)	electrolyte/membrane	back electrode	potential range	ε_max_	ε_min_	Δε/ε
CVD graphene^16^	≈35	[DEME][TFSI]/PE	gold	0 to +3 V	0.8	0.3	0.63
CVD graphene^18^	120–150	[BMIM][PF_6_]/fabric-cotton	gold	0 to +6 V	0.7	0.35	0.50
CVD graphene^17^	≈40	battery grade LiPF_6_ in organic/PE	Li-NMC/on Al	0 to +3.8 V	0.7	0.25	0.64
rGO^19^	n/a	[BMIM][PF_6_]/PS filter membrane	gold	0 to +5 V	0.77	0.6	0.22
MWCNTs^20^ (drawn forest)	≈0.5	[HMIM][NTf]/PE	MWCNTs	–1.7 to 3 V	0.7	0.15	0.79
SWCNTs (this work)	≈40	[DEME][TFSI]/PE	SWCNTs	–1 to 2.5 V	0.54	0.24	0.56

## Conclusions

In this paper, we demonstrate solution-processable carbon nanotube networks on electrolyte-infused membranes with the ability to switch transparency in the MIR. The devices also exhibit modulation of emissivity when mounted on a surface. Understanding of the performance is developed through examining the relationship between electronic and optothermal properties. The properties of the devices can be tuned with SWCNT layer thickness to optimize the characteristics. Particularly, since at high film thicknesses, both maximum emissivity and relative emissivity change are low. The understanding developed from infrared spectroscopy helps inform device design, allowing for switching between emissivity of 0.54 to 0.24. The device is fully characterized in terms of stability and performance. Due to the simplicity of processing of this material, it is believed that the devices can be scaled arbitrarily with advances in nanomaterial printing technologies. Scalability of this functionality could be instrumental to the development of sustainable heat management technologies.

## Materials and Methods

### Materials

SWCNTs and TUBALL BATT H_2_O (battery grade, diameter < 2 nm, 0.4 wt % in water with 0.8 wt % sodium carboxymethyl cellulose, CMC, binder) were purchased from OCSiAl. Diethylmethyl(2-methoxyethyl)ammonium bis(trifluoromethylsulfonyl)imide [DEME][TFSI] was purchased from Merck. Polyethlyene (PE) membranes were provided by Entek. Deionized water (18.2 MΩ·cm resistivity) was produced with a Thermo Scientific Barnstead MicroPure purification system.

### Preparing Inks for Spraying

SWCNTs were taken as supplied and diluted by a factor of 10 in deionized water and sonicated in a bath for 10 min.

### Preparing Devices

For the SWCNT devices, the PE membrane was infiltrated with the ionic liquid by adding 100 μL per cm^2^ and letting it soak for 10 min. The previously prepared dispersion was spray-deposited with an airbrush at 3 bar at a distance of 15 cm through an appropriate stencil. The amount of material deposited was controlled by the volume of dispersion sprayed onto the substrate. The area was approximately 50 cm^2^, and the volumes were in the range of 0.5–10 mL. This was done on a hotplate at 90 °C. No attempt to remove the carboxymethyl cellulose stabilizer was made to facilitate a straight-out-of-the-bottle approach. Electrical connection to the potentiostat was made through copper tape. Sheet resistance of the films was measured using a sacrificial piece of film on the substrate and the transmission line method.

### Thermal Measurements

Thermal measurements were performed using a FLIR 4C thermal camera (spectral range 8–14 μm, accuracy +/– 3 °C, the factory calibration was used and confirmed using glass substrate ε ≈ 0.96 and sputtered gold ε ≈ 0.07) placed 30 cm above the devices which were mounted on a hotplate. Temperature was allowed to stabilize for 5 minutes before measurements as observed by a thermal camera. A Gamry 600+ potentiostat was used to provide the potential bias in all experiments.

### Raman Spectroscopic Measurements

Raman spectroscopic measurements were performed using a Renishaw inVia Qontor spectrometer with 488 nm (2.54 eV), 532 nm (2.33 eV), 660 nm (1.88 eV), and 785 nm (1.58 eV) laser sources. Acquisition powers ranged from 4–7 mW with 10 accumulations of 10 s.

### UV–Vis Measurements

The UV–vis measurements of dispersions were performed with a Shimadzu UV-3600 Plus spectrophotometer from 300–800 nm using a plastic cuvette (path length, 1 cm). These data were used to stitch to NIR of deposited films. Liquid-based data was used to film thickness effects as general trends in absorption were observable in liquid.

### FTIR Measurements

The FTIR measurements were performed using a PerkinElmer Frontier FTIR/NIR spectrometer. All of the measurements were performed in the mid-IR spectral range (7800–400 cm^–1^, the optimum scan range) in transmission mode. Thin-film NIR (15000–2000 cm^–1^, the optimum scan range) was also performed for stitching to give a broader picture of the CNT film optical density or −log(*T*). Stitching in Figure 3a was simply done by matching absorbance at the edge of each respective spectrum by normalization.

### AFM Imaging

The Dimension Icon system from Bruker operating in the PeakForce quantitative nanoscale mechanical (QNM) mode was used for atomic force microscopy (AFM) measurements. The probe used was a ScanAsyst Air tip with a spring constant of 0.4 N m^–1^, and a tip–sample contact force of 5.0 nN was used for all measurements.

### SEM Imaging

SEM imaging was performed with a Zeiss SIGMA field emission gun scanning electron microscope (FEG-SEM) using a Zeiss in-lens secondary electron detector. The FEG-SEM working conditions used were 2.5 kV accelerating voltage, 20 μm aperture, and 2 mm working distance.

## Supporting Information Available

AFM and Raman characterization of carbon nanotubes; additional electrochemical measurements; and FTIR spectroscopy measurements and emissivity fits for all devices (PDF)
