# Supplementary material for: Mid-Infrared Electrochromics Enabled by Intraband Modulation in Carbon Nanotube Networks
Source: ACS Appl Mater Interfaces. 2023 Feb 17;15(8):11225–33. doi: 10.1021/acsami.2c19758 (PMC9982807; doi:10.1021/acsami.2c19758)
Supplement: Supplementary file 1 — am2c19758_si_001.pdf [file am2c19758_si_001.pdf]

Supporting Information: Mid-infrared electrochromics enabled by intraband modulation in carbon nanotube networks

Peter J. Lynch[1]*, Manoj Tripathi[1], Aline Amorim Graf[1], Sean P. Ogilvie[1], Matthew J. Large[1], Jonathan P. Salvage[2], Alan B. Dalton[1]*

[1] University of Sussex, Department of Physics and Astronomy, Brighton, BN1 9RH, UK

[2] University of Brighton, School of Pharmacy and Biomolecular Science, Brighton, BN2 4GJ, UK

*p.j.lynch@sussex.ac.uk

* a.b.dalton@sussex.ac.uk

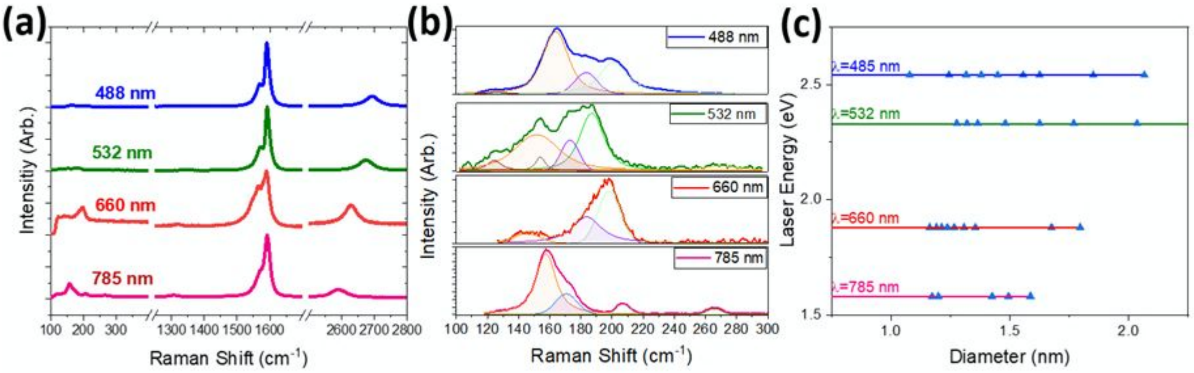

Figure S1 (a) Full Raman spectrum and (b) RBM region with peak fittings to extract diameter of the deposited nanotubes on Si at various laser energies. (c) Estimated diameter from fitting RBM peaks for laser lines of 488, 532, 660 and 785 nm plotted as a Kataura Plot.

Figure S1(a) shows Raman spectra of the nanotube films using various laser lines. The radial breathing modes are plotted in figure S1(b) and fitted to show the contributing peaks. The observed estimated diameters from the radial breathing modes Raman shift were extracted using the formula $\omega_{RBM} = A/d_t + B$ where ω_{RBM} is the shift of the peak corresponding to a particular tubes radial breathing mode, d_t is the diameter of the carbon nanotubes, and A and B are constants[1]. These diameters can be super-imposed on the Kataura plot[2] for each relevant laser line. Due to the effect of the bundle shift on

observed diameter as well as the inability of the laser line to be exactly in resonance, the points often do not match up exactly with a particular point on the Kataura plot. To this end, the plot can be used qualitatively.

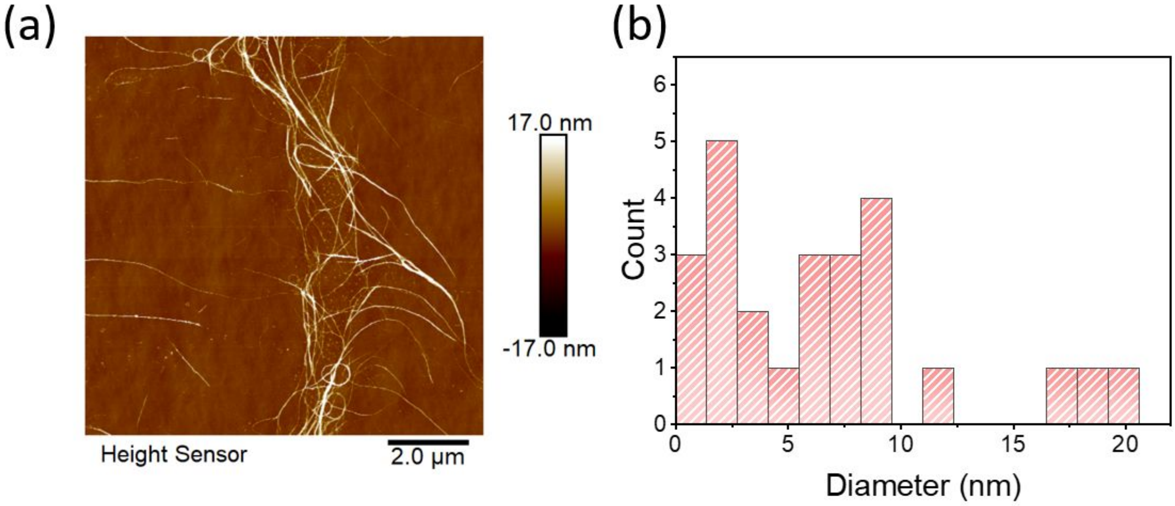

Figure S2 (a) AFM micrograph of dropcasted CNTs on Si. (b) Corresponding AFM statistics of tube diameter.

Analysis of the AFM in figure S2a allowed to look at individual and bundled tubes. The diameters vary from low (indicating single tubes) to large indicating bundles. AFM image shows variation in nanotubes diameter from large bundles up to 20 nm in diameter to an individualised nanotube of

diameter around 0.5 nm. The statistical distribution of different diameters illustrated through histogram in figure S2b.

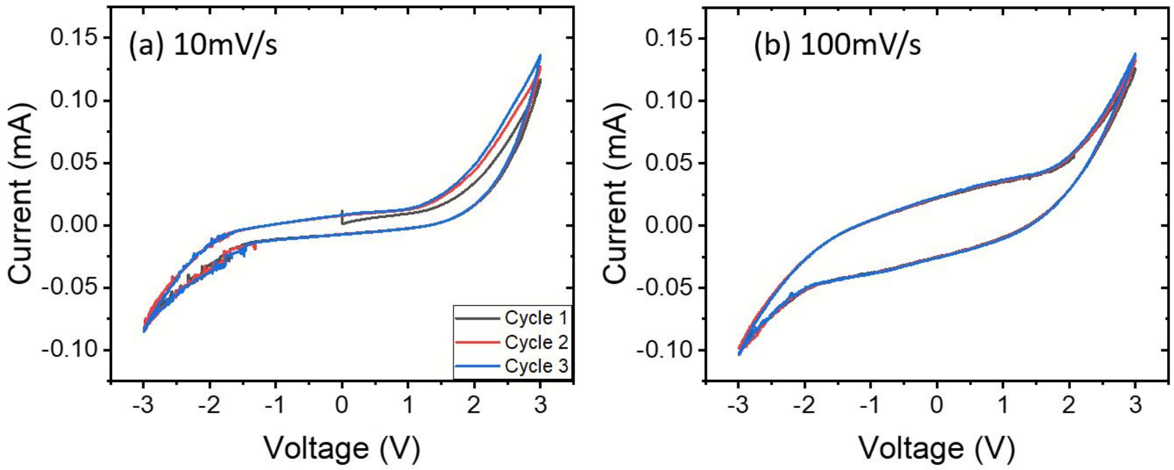

Figure S3 Cyclic voltammetry of 70 Ω/□ device at 100 °C at (a) 10 mV/s for 3 cycles and (b) 100 mV/s for 3 cycles.

Cyclic voltammetry of the device at elevated temperature (100 °C) shows drift in the current above 2 V with each cycle at low scan rates (10 mV/s, figure 2a) this is less observable at higher scan rates (100 mV/s, figure 2b) suggesting this is a faradaic process. These processes could be degradation of the electrolyte due to electrolysis or the removal of water through electrolysis. Since the current is

increasing it is more likely to be electrolyte degradation as the current from hydrolysis should decrease as the cycles progress. The 3 cycles were produced after 20 cycles at 100 mV/s.

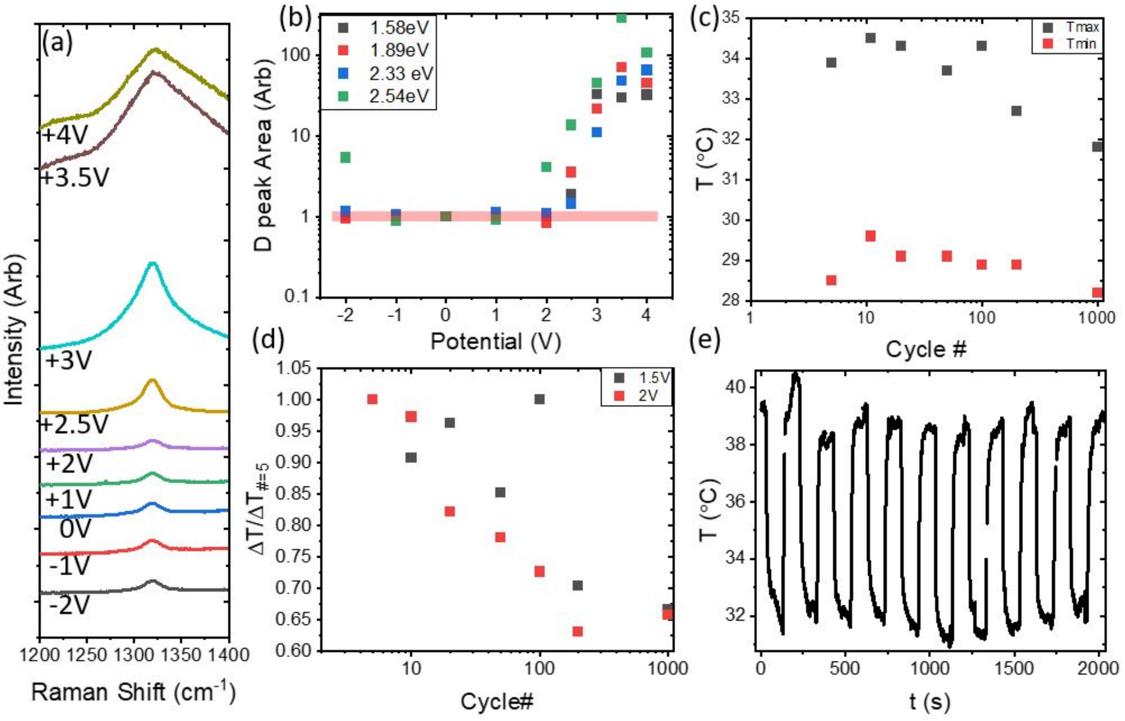

Figure S4 Stability considerations of the symmetric semiconducting device. (a) The D-band of the nanotubes after the device has been onto the indicated potential for 5 minutes. Spectra were then taken of the unbiased devices. (b) Integrated D peak area for a range of different laser lines after biasing at various potentials. (c) Maximum and minimum temperature of the device cycled at 2 V. (d) Change in temperature difference for devices biased at +1.5 and + 2 V, respectively. (e) Sample of 10 cycles of the device.

The influence of the different potential bias on nanotubes electrode were investigated through Raman spectroscopy by monitoring changes in the D-peak (≈1322 cm^{-1}). The higher intensity of D-peak indicates an increasing number of defects in the graphitic structure. [3] Figure S4a shows the increment in the D-peak intensity and its gradual broadening with increasing biases. Qualitatively, The D-peak undergoes a significant increase in intensity, width (FWHM), and background scattering after exceeding +2.5 V. The broad peak features of D-peak feature are associated with the presence of amorphous carbon,[4] revealing the transformation of some sp^2 carbon atoms into sp^3 through structural disorder. It indicates electrode damage through the amorphization of carbon structure under electrical stimulation with a threshold voltage of +2.5 volts. This trend is reproducible with several laser energy (1.58 to 2.54 eV), figure S4b). Interestingly, the higher energy laser shows more instability which corresponds to the semiconducting tubes of lower diameter. With the consideration of cyclability of devices, the performance was monitored through cycles between -1 V and 1.5 or 2 V for 1000 cycles. They show reasonable stability with comparable effective temperature drop to that of the device at optimum performance. Figures S4c, d and e characterise this stability showing a drop in the maximum observed temperature (T_{max}) with cycle number as well as a smaller drop in minimum observed temperature (T_{min}). The overall fraction of the device performance after 1000 cycles over the initial cycle was approximately 0.65".

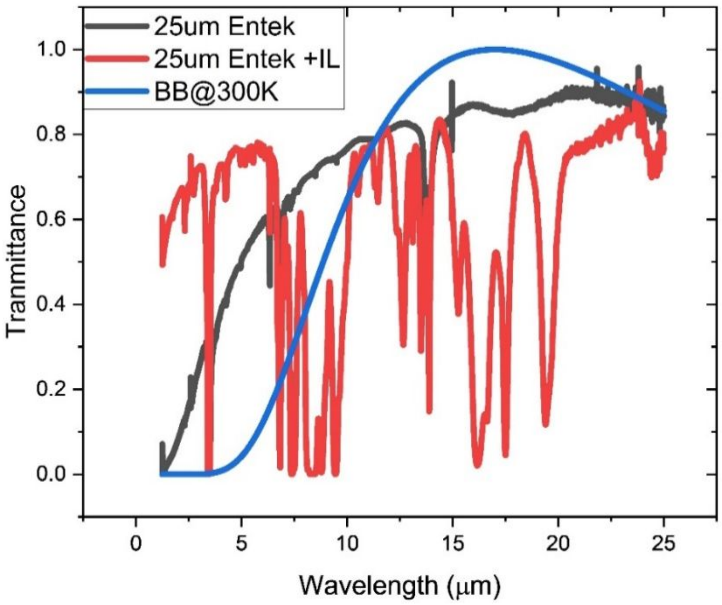

Figure S5 FTIR spectra of 25-micron Entek membrane, 25-micron Entek membrane infiltrated with ionic liquid [DEME][TFSI]. Blue line is black body emission at 300 K.

FTIR spectrum of PE (black) showing largely transparent film at high wavelengths with a reduction in transparency at lower wavelengths due to scatter induced by the nature of the porous membrane. The typical PE lines are observed. Once soaked with the [DEME][TFSI] ionic liquid the scattering component is reduced due to index matching of the filled pores and additional peaks due to the ionic liquid are observed. Black body radiation at 300 K added as reference to illustrate the spectral radiation of bodies in the temperature range of interest, and it’s overlap in the spectral range of the devices studied.

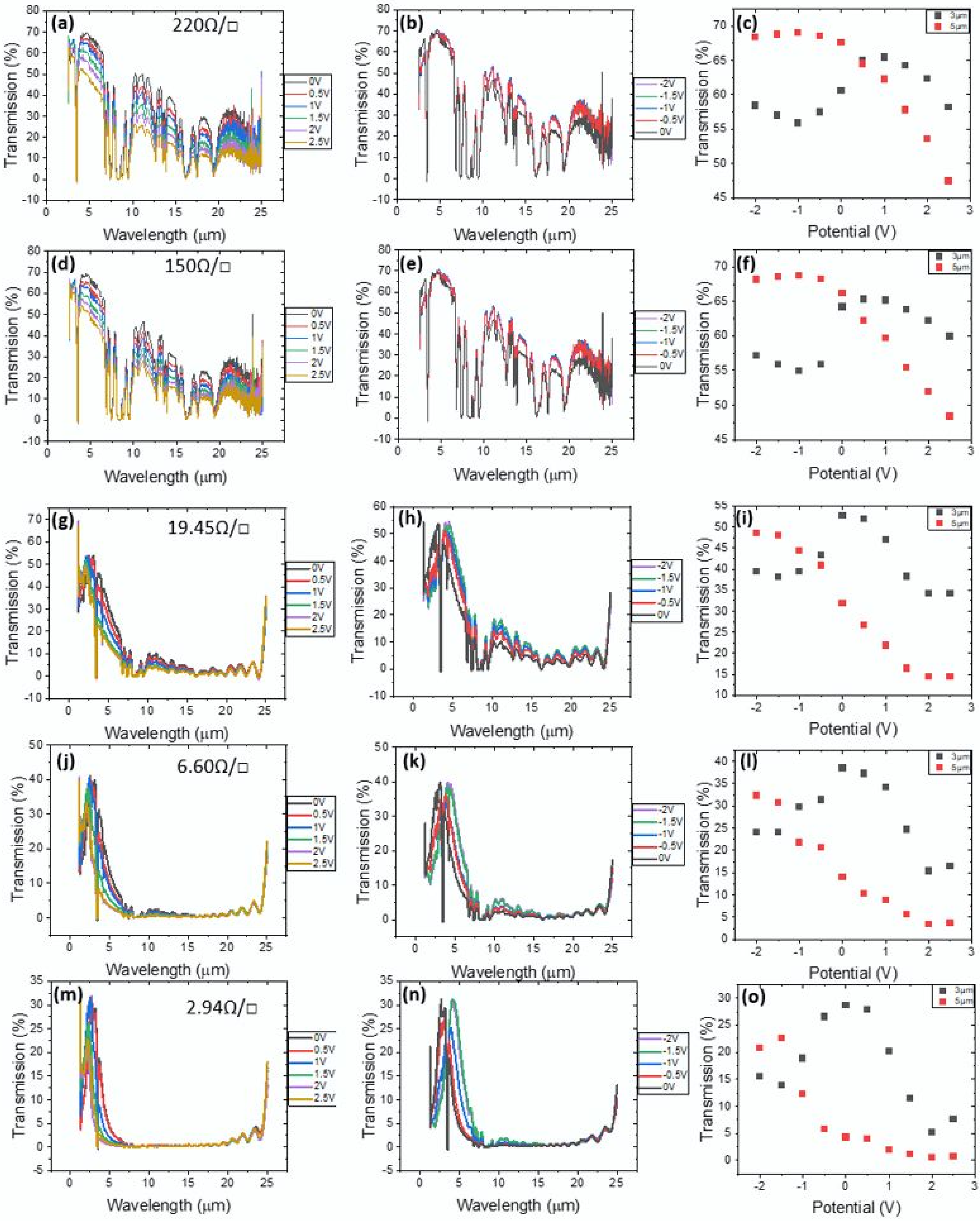

Figure S6 (a, b, c) FTIR spectra positive biased, negative biased single film devices and transmission at 3- and 5-micron thickness for films with sheet resistance of 220 Ω/□. (d,e,f) Similarly for devices of 150 Ω/□, (g,h,i) 19.5 Ω/□, (j,k,l) 6.6 Ω/□, and (m,n,o) 2.94 Ω/□.

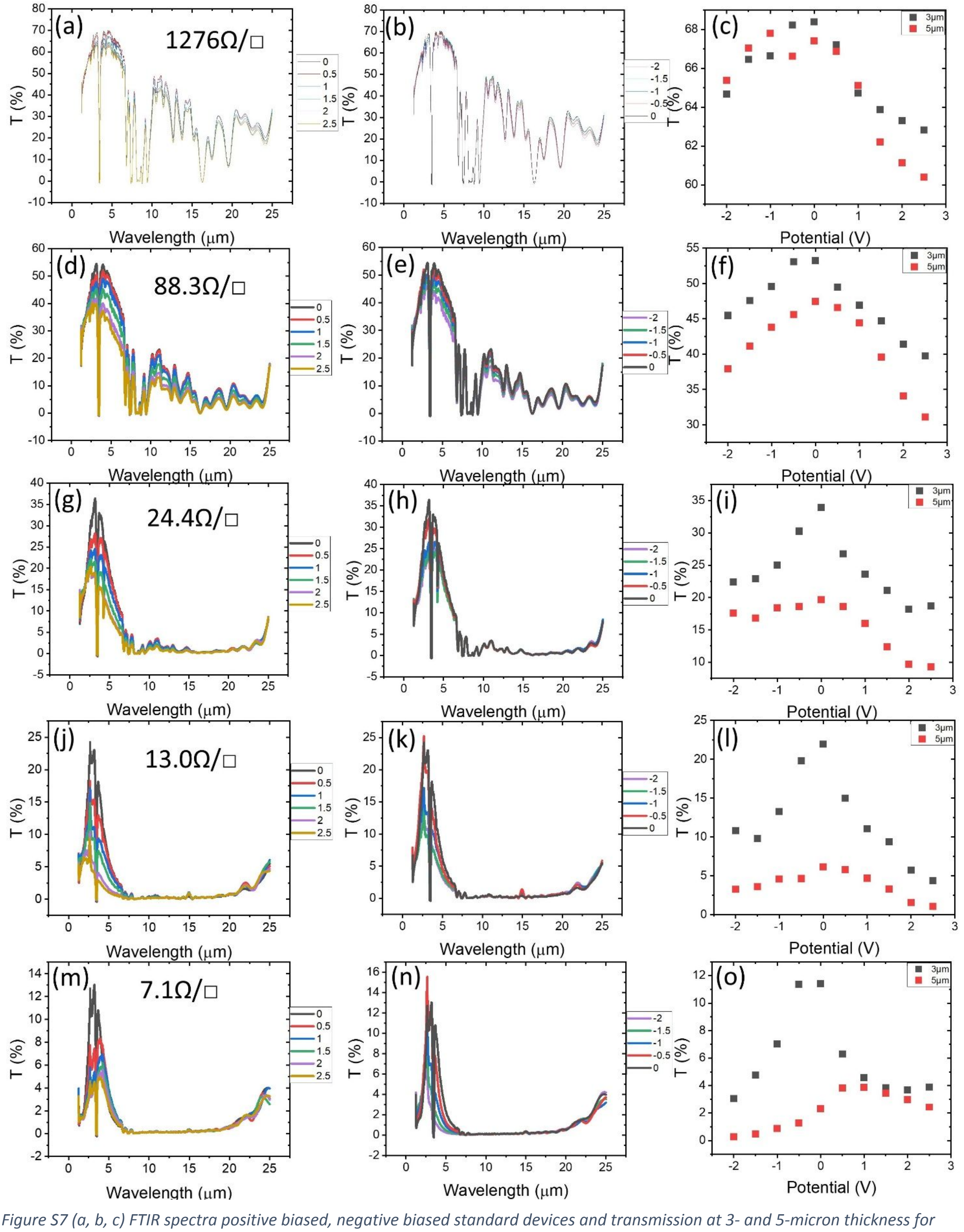

Figure S7 (a, b, c) FTIR spectra positive biased, negative biased standard devices and transmission at 3- and 5-micron thickness for films with sheet resistance of 1276 Ω/□. (d,e,f) Similarly for devices of 88.3 Ω/□, (g,h,i) 24.4 Ω/□, (j,k,l) 13.0 Ω/□, and (m,n,o) 7.1 Ω/□.

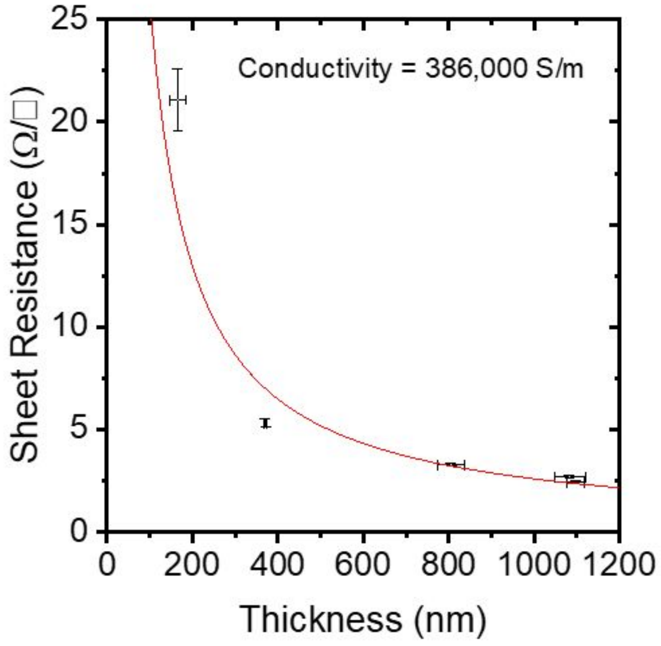

Figure S8 Sheet Resistance of carbon nanotube films of varying thicknesses, with a 1/t fit to estimate the conductivity at 386,000 S/m

5 films were sprayed on a glas substrate to provide insight to the behaviour of sheet resistance with thickness. Which is $\sigma_{DC} = 1/(R_s t)$. The fit of the data indicates a conductivity of 386,000 +/- 19,000 S/m.

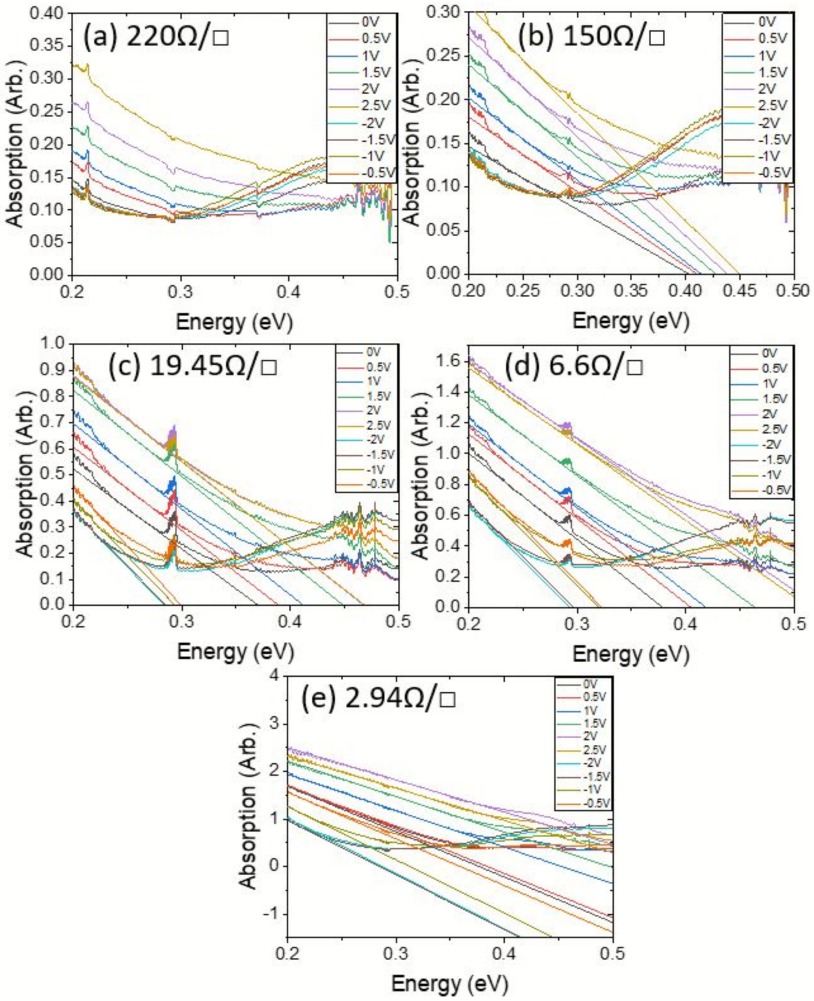

Figure S9(a) Absorption vs. *energy for 220 Ω/□ single film device at various biases from -2 to +2.5 V. (b) Absorption* vs. *energy for 150 Ω/□ single film device at various biases from -2 to +2.5 V with fitting linear portion of curve to extrapolate band edge for positive biases. (c,d,e) Absorption* vs. *energy for 19.45, 6.6 and 2.94 Ω/□ single film devices respectively with corresponding extrapolation to band edge for all potential biases.*

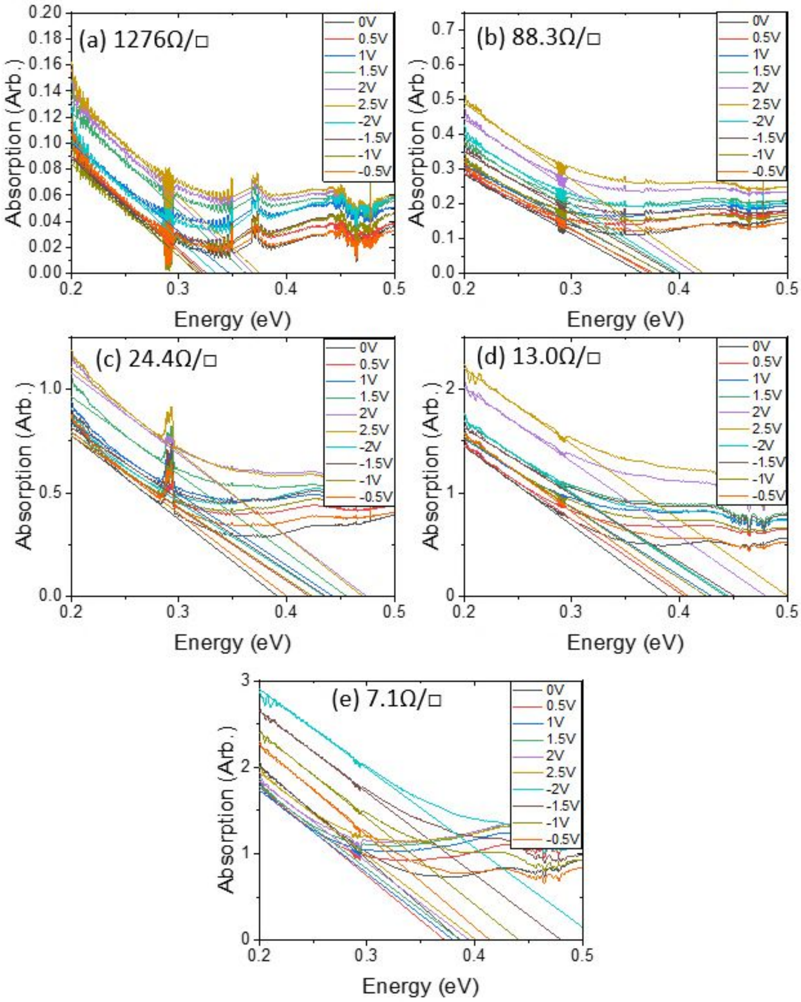

Figure S10(a) Absorption vs. *energy for 1276 Ω/□ single film device at various biases from -2 to +2.5 V. (b) Absorption* vs. *energy for 88.3 Ω/□ single film device at various biases from -2 to +2.5 V with fitting linear portion of curve to extrapolate band edge for positive biases. (c,d,e) Absorption* vs. *energy for 24.4, 13.0 and 7.1 Ω/□ single film devices respectively with corresponding extrapolation to band edge for all potential biases.*

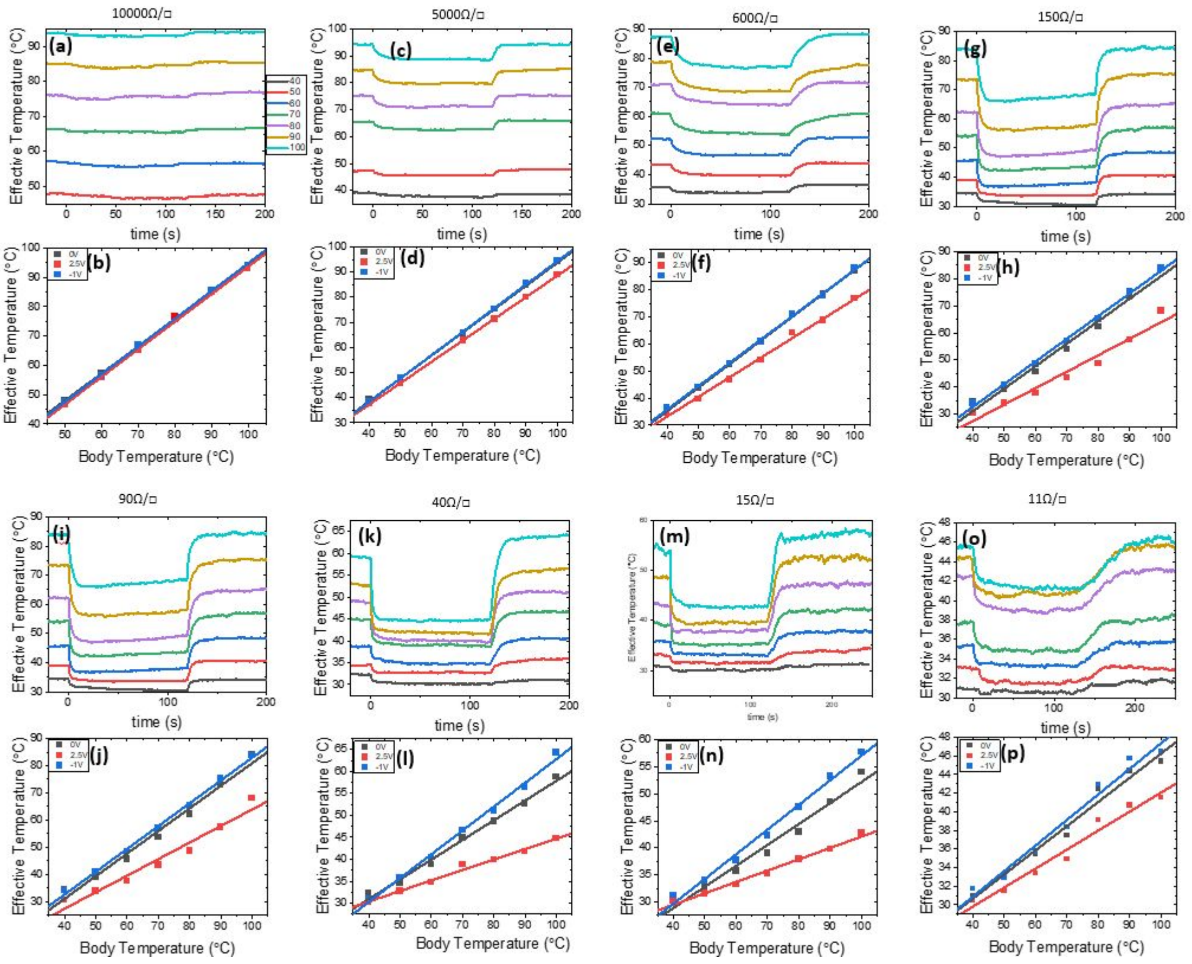

Figure S11 (a,b) Temperature vs. time and effective temperature vs. body temperature of device at 10,000 Ω/□, (c,d) 5,000 Ω/□, (e,f) 600 Ω/□, (g,h) 150 Ω/□, (i,j) 90 Ω/□, (k,l) 40 Ω/□, (m,n) 15 Ω/□, and (o,p) 11 Ω/□.

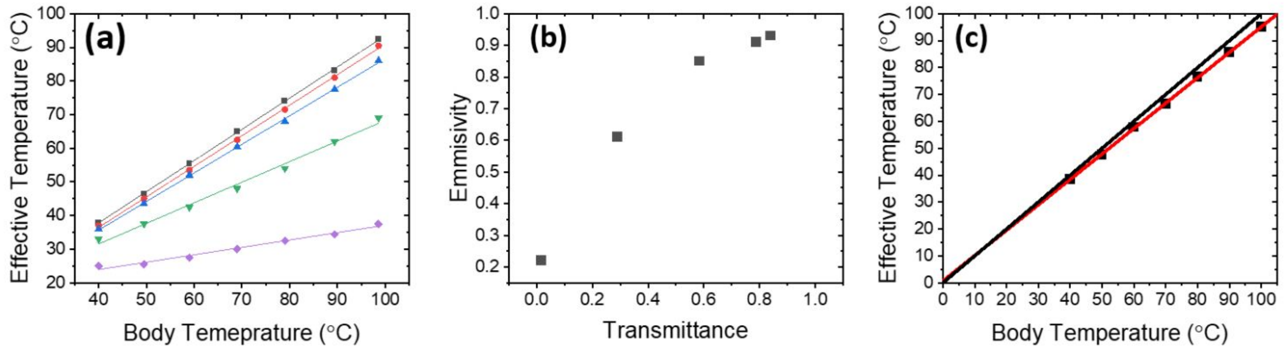

Figure S12(a) Fittings of the temperature from a thermal camera versus the sample temperature for various thicknesses of carbon nanotubes on PET with fit to extract the effective emissivity (b) The effective emissivity of the CNT films on PET plotted against optical transparency at 600 nm, (c) The apparent vs. *body temperature plot for the PET substrate.*

Figure S12(a) plots the effective temperature of sprayed SWCNT films on PET against the body temperature and Figure S12(b) plots the emissivity of the films against the optical transparency at 550 nm, the more opaque in the visible, the lower the emissivity in the IR for the SWCNT films. Figure S12(c) shows the effective temperature of the PET substrate against body temperature and it is worth noting that the emissivity in Figure S11b tends towards the emissivity of the substrate. Therefore, the transparency of the device, as well as the changes in transparency in Figure 4 contribute to the effective emissivity of the device. These devices operated in air maintain approximately 66% of the temperature differential over 1000 cycles (figure S3).

(1) Jorio, A.; Pimenta, M. A.; Filho, A. G. S.; Saito, R.; Dresselhaus, G.; Dresselhaus, M. S. Characterizing Carbon Nanotube Samples with Resonance Raman Scattering. *New J. Phys.* **2003**, *5*, 139–139. https://doi.org/10.1088/1367-2630/5/1/139.

(2) Kataura, H.; Kumazawa, Y.; Maniwa, Y.; Umezu, I.; Suzuki, S.; Ohtsuka, Y.; Achiba, Y. Optical Properties of Single-Wall Carbon Nanotubes. *Synth. Met.* **1999**, *103* (1–3), 2555–2558. https://doi.org/10.1016/S0379-6779(98)00278-1.

(3) Bacsa, R. R.; Flahaut, E.; Laurent, C.; Peigney, A.; Aloni, S.; Puech, P.; Bacsa, W. S. Narrow Diameter Double-Wall Carbon Nanotubes: Synthesis, Electron Microscopy and Inelastic Light Scattering. *New J. Phys.* **2003**, *5*, 131–131. https://doi.org/10.1088/1367-2630/5/1/131.

(4) Ferrari, A. C.; Robertson, J. Interpretation of Raman Spectra of Disordered and Amorphous Carbon. *Phys. Rev. B* **2000**, *61* (20), 14095–14107. https://doi.org/10.1103/PhysRevB.61.14095.
